# Supplementary material for: CD1d-dependent rewiring of lipid metabolism in macrophages regulates innate immune responses
Source: Nat Commun. 2022 Nov 7;13:6723. doi: 10.1038/s41467-022-34532-x (PMC9640663; doi:10.1038/s41467-022-34532-x)
Supplement: Supplementary file 1 — Supplementary Information [file 41467_2022_34532_MOESM1_ESM.pdf]

SUPPLEMENTARY INFORMATION

CD1d-dependent rewiring of lipid metabolism in macrophages regulates innate immune responses

Phillip M. Brailey, Lauren Evans, Juan Carlos López-Rodríguez, Anthony Sinadinos, Victoria Tyrrel, Gavin Kelly, Valerie O'Donnell, Peter Ghazal, Susan John & Patricia Barral

This PDF file includes Supplementary Figures 1 to 6 and Supplementary Table 1

Supplementary Figure 1

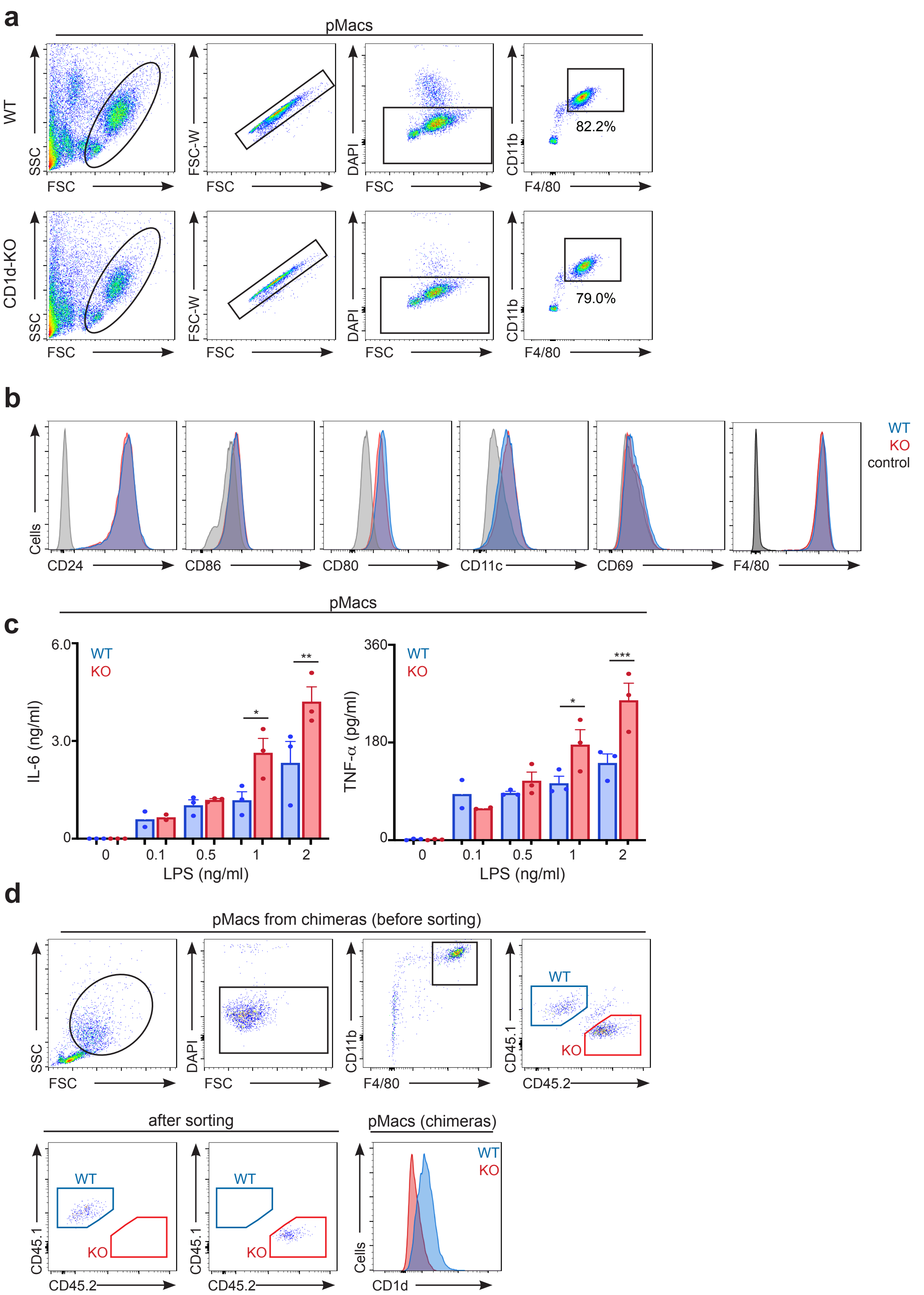

Supplementary Figure 1. Characterization of WT and CD1d-KO pMacs. **(a)** Flow cytometry profile showing populations of WT and CD1d-KO pMacs. **(b)** Representative flow cytometry for WT (blue) and CD1d-KO (red) pMacs showing expression of the depicted markers. **(c)** Secretion of IL-6 (left) and TNF-α (right) in WT and CD1d-KO pMacs stimulated with LPS at the indicated concentrations (n=2-3). Bars represent mean +/- SEM; *p<0.05; **p<0.01, ***p<0.001, 2-way ANOVA. **(d)** Flow cytometry profiles showing gating strategy for purification of WT and CD1d-KO pMacs from BM chimeras. Values for n represent biologically independent samples (as shown by the number of data points in each graph). n= cells isolated from individual mice. Source data are provided as a Source Data file

Supplementary Figure 2

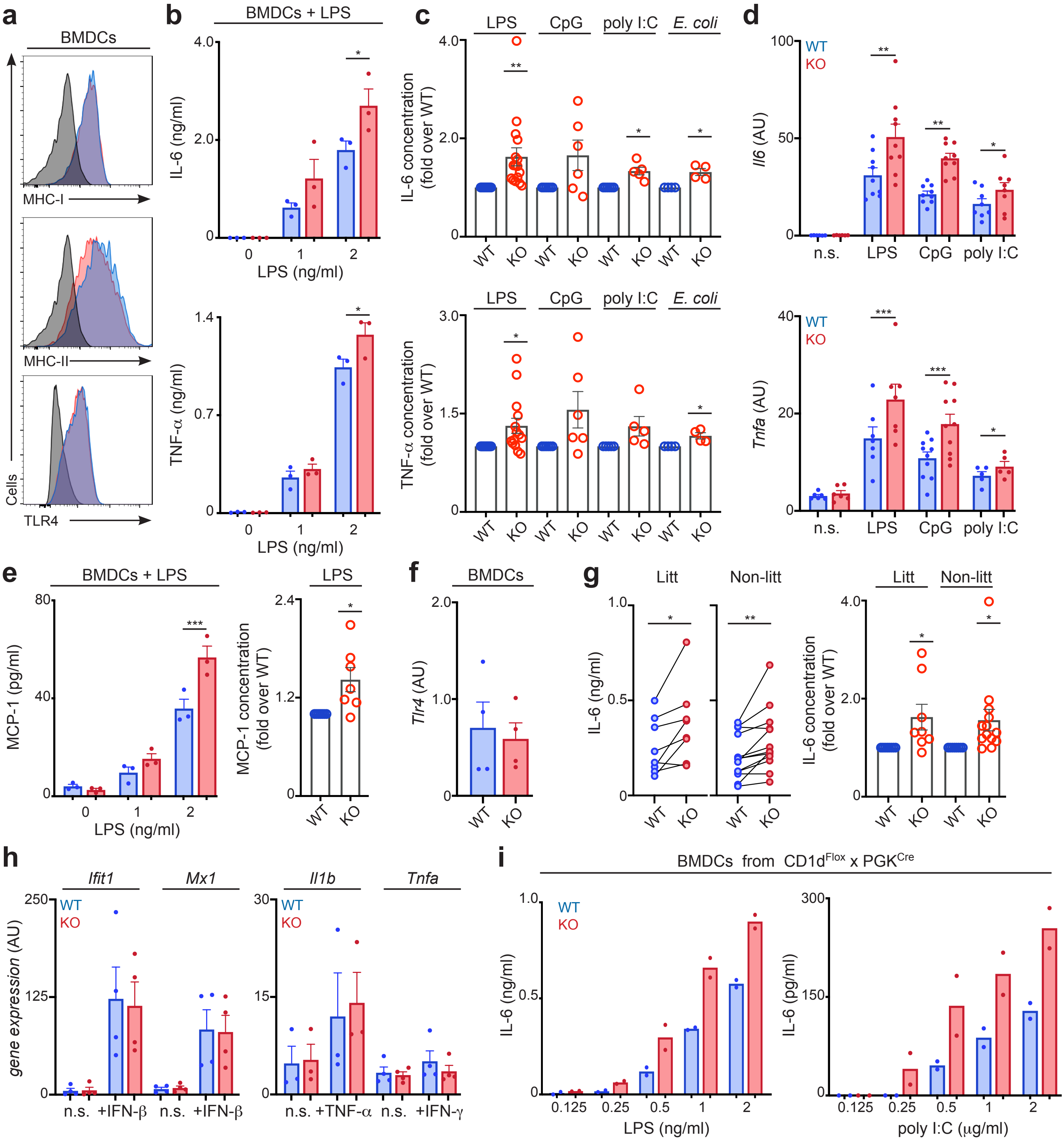

Supplementary Figure 2. CD1d functions as a negative regulator of TLR responses
(a) Representative flow cytometry of WT (blue) and CD1d-KO (red) BMDCs. **(b)** Cytokine secretion by WT (blue) and CD1d-KO (red) BMDCs stimulated with LPS as indicated (n=3). *p<0.05; 2-way ANOVA. **(c-d)** Normalised concentration of secreted cytokines (fold over WT, c) and gene expression (d) in WT and CD1d-KO BMDCs cultured with the depicted stimuli for 6h (n=4-16). *p<0.05; **p<0.01; ***p<0.001 one-sample (c) or paired two-tailed t-test (d). n.s.=non stimulated. **(e)** Secretion of MCP-1 (left, n=3) and normalised concentration of MCP-1 (fold over WT, right, n=7) for BMDCs after stimulation with LPS. *p<0.05; ***p<0.001; 2-way ANOVA (left), or one-sample t-test (right). **(f)** qPCR expression data of *Tlr4* in WT and CD1d-KO BMDCs (n=4). **(g)** Secretion of IL-6 (left) and normalised concentration of IL-6 (fold over WT, right) for BMDCs after stimulation with LPS as above (n=8-13). Cells were either isolated from littermate controls (Litt) or from age and sex matched WT and CD1d-KO mice (Non-Litt). *p<0.05; **p<0.01; two-tailed paired (left) or one-sample t-test (right). **(h)** Expression of the depicted genes in WT and CD1d-KO BMDCs after stimulation with IFN-β, TNF-α or IFN-γ (n=4). n.s.=non stimulated. **(i)** WT or CD1d-KO BMDCs were generated from CD1dFlox x PGKCre mice and stimulated with TLR ligands. IL-6 secretion was measured by ELISA. Data are representative results pooled from 2 experiments. Bars in all graphs represent mean +/- SEM. Values for n represent biologically independent samples (as shown by the number of data points in each graph). n= cells isolated from individual mice. Source data are provided as a Source Data file

Supplementary Figure 3

Down in KO

id	log2FoldChange	symbol
ENSMUSG00000078377	-29.02687683	
ENSMUSG00000082141	-25.51405833	
ENSMUSG00000039252	-25.17376208	Lgi2
ENSMUSG00000065870	-25.15593314	
ENSMUSG00000101952	-24.49460803	
ENSMUSG00000096474	-24.38033012	
ENSMUSG00000067736	-23.19460402	
ENSMUSG00000081911	-22.4070127	
ENSMUSG00000046952	-21.80931864	
ENSMUSG00000045587	-21.7572145	BC049730
ENSMUSG00000082087	-21.50619188	
ENSMUSG00000092463	-21.00054731	
ENSMUSG00000071141	-20.16127284	
ENSMUSG00000068397	-19.92045506	
ENSMUSG00000084383	-19.92045506	
ENSMUSG00000030804	-19.84615886	
ENSMUSG00000053916	-19.58641821	Nanp
ENSMUSG00000010136	-19.53756849	Pifo
ENSMUSG00000094737	-19.40188341	
ENSMUSG00000029419	-19.35780074	Gm996
ENSMUSG00000083274	-19.28172668	
ENSMUSG00000040666	-19.19481094	Sh3bgr
ENSMUSG00000105286	-19.17992727	
ENSMUSG00000030546	-19.15470464	Plin1
ENSMUSG00000110471	-19.14782716	
ENSMUSG00000085791	-19.142776	
ENSMUSG00000097060	-19.13868066	
ENSMUSG00000090707	-19.12414804	
ENSMUSG00000037846	-19.08291678	Rtkn2
ENSMUSG00000068962	-18.48410958	Zfp114
ENSMUSG00000084241	-18.47966616	
ENSMUSG00000066007	-18.47402191	Zfp600
ENSMUSG00000049908	-18.33106189	Gja8
ENSMUSG00000025272	-18.32751133	Tro
ENSMUSG00000081529	-18.25436017	
ENSMUSG00000031876	-18.23381501	
ENSMUSG00000045794	-18.19147542	Lkaaear1
ENSMUSG00000092405	-18.15286756	
ENSMUSG00000090785	-18.02278912	
ENSMUSG00000061742	-18.01451198	Slc22a12
ENSMUSG00000087951	-18.01195064	
ENSMUSG00000083038	-18.01130463	
ENSMUSG00000092345	-17.92963999	
ENSMUSG00000083310	-17.84500115	
ENSMUSG00000093574	-17.8365345	
ENSMUSG00000066491	-17.78414861	
ENSMUSG00000035131	-17.77972288	Brinp3
ENSMUSG00000094271	-17.68592972	Gm13290
ENSMUSG00000087654	-17.63237787	
ENSMUSG00000104608	-17.55753549	
ENSMUSG00000064194	-17.52080742	Zfp936
ENSMUSG00000105835	-17.47650905	
ENSMUSG00000042874	-17.38238539	
ENSMUSG00000084836	-17.36237098	
ENSMUSG00000089621	-17.33463081	
ENSMUSG00000101867	-17.31038224	
ENSMUSG00000106090	-17.30617168	
ENSMUSG00000101459	-17.30611952	
ENSMUSG00000092232	-17.2858375	
ENSMUSG00000065406	-17.27267003	Mirlet7i
ENSMUSG00000082318	-17.23789548	
ENSMUSG00000092389	-17.2374632	
ENSMUSG00000061626	-17.23736133	Olfr68
ENSMUSG00000093720	-17.22543197	
ENSMUSG00000057766	-17.2004269	Ankrd29
ENSMUSG00000032023	-17.11337011	Jhy
ENSMUSG00000086384	-17.081537	
ENSMUSG00000092760	-17.05429563	
ENSMUSG00000086827	-17.02719809	
ENSMUSG00000050425	-17.02713507	Mrgprb2
ENSMUSG00000083708	-16.66318016	
ENSMUSG00000040473	-16.46604263	Cfap69
ENSMUSG00000082195	-16.00229983	
ENSMUSG00000078963	-16.00091701	Hsbp1l1
ENSMUSG00000077709	-15.50324552	Snora64
ENSMUSG00000028076	-5.502108487	Cd1d1
ENSMUSG00000063458	-1.724529128	Lrmda
ENSMUSG00000028081	-1.638195772	Rps3a1
ENSMUSG00000004891	-1.339610277	Nes
ENSMUSG00000025150	-1.226347434	Cbr2
ENSMUSG00000015568	-1.20797248	Lpl
ENSMUSG00000032725	-1.097863983	Folr2
ENSMUSG00000026675	-1.026861817	Hsd17b7
ENSMUSG00000021508	-0.851522988	Cxcl14
ENSMUSG00000000489	-0.792243357	Pdgfb
ENSMUSG00000031373	-0.77861091	Car5b
ENSMUSG00000066632	-0.772226994	
ENSMUSG00000032786	-0.767240341	Alas1
ENSMUSG00000032561	-0.709662034	Acpp
ENSMUSG00000027940	-0.695303653	Tpm3
ENSMUSG00000019302	-0.682497034	Atp6v0a1
ENSMUSG00000098557	-0.670531542	Kctd12
ENSMUSG00000002992	-0.659858141	Apoc2
ENSMUSG00000038540	-0.646951734	Tmc3
ENSMUSG00000058258	-0.634808577	Idi1
ENSMUSG00000017754	-0.63377792	Pltp
ENSMUSG00000059743	-0.597290601	Fdps
ENSMUSG00000037536	-0.58241735	Fbxo34
ENSMUSG00000031827	-0.581882905	Cotl1
ENSMUSG00000037868	-0.569863422	Egr2
ENSMUSG00000001467	-0.565282341	Cyp51
ENSMUSG00000079293	-0.516240515	Clec7a
ENSMUSG00000093930	-0.483777701	Hmgcs1
ENSMUSG00000032018	-0.483071462	Sc5d
ENSMUSG00000028691	-0.476256898	Prdx1
ENSMUSG00000021273	-0.448175672	Fdft1
ENSMUSG00000031604	-0.446118195	Msmo1
ENSMUSG00000040451	-0.444125925	Sgms1
ENSMUSG00000032462	-0.40813545	Pik3cb
ENSMUSG00000045294	-0.379533015	Insig1

Up in KO

id	log2FoldChange	symbol
ENSMUSG00000020680	0.385143837	Taf15
ENSMUSG00000061175	0.444649512	Fnip2
ENSMUSG00000001751	0.471676243	Naglu
ENSMUSG00000037260	0.486891168	Hgsnat
ENSMUSG00000025453	0.530735068	Nnt
ENSMUSG00000049866	0.537291735	Arl4c
ENSMUSG00000055172	0.611361153	C1ra
ENSMUSG00000028086	0.645631736	Fbxw7
ENSMUSG00000038034	0.663384004	Igsf8
ENSMUSG00000004709	0.668737665	Cd244
ENSMUSG00000044468	0.67018404	Fam46c
ENSMUSG00000086503	0.670549626	Xist
ENSMUSG00000024030	0.714374305	Abcg1
ENSMUSG00000028015	0.731429996	Ctso
ENSMUSG00000073643	0.736013728	Wdfy1
ENSMUSG00000000958	0.737899591	Slc7a7
ENSMUSG00000032204	0.740203525	Aqp9
ENSMUSG00000025408	0.754618174	Ddit3
ENSMUSG00000001281	0.765637973	Itgb7
ENSMUSG00000023078	0.766462641	Cxcl13
ENSMUSG00000032216	0.808795868	Nedd4
ENSMUSG00000008734	0.861510092	Gprc5b
ENSMUSG00000034271	0.888884445	Jdp2
ENSMUSG00000028064	0.975414951	Sema4a
ENSMUSG00000037443	0.97602546	Cep85
ENSMUSG00000032715	0.982518973	Trib3
ENSMUSG00000031078	1.128483882	Cttn
ENSMUSG00000004359	1.156690765	Spic
ENSMUSG00000027313	1.183963175	Chac1
ENSMUSG00000027799	1.227967105	Nbea
ENSMUSG00000024011	1.288366405	Pi16
ENSMUSG00000059994	1.292605881	Fcrl1
ENSMUSG00000040690	1.32644391	Col16a1
ENSMUSG00000052477	1.32836733	C130026I21Rik
ENSMUSG00000034205	1.362038476	Loxl2
ENSMUSG00000027962	1.498433165	Vcam1
ENSMUSG00000038085	1.622206448	Cnbd2
ENSMUSG00000045573	1.660739418	Penk
ENSMUSG00000079190	1.871276669	LOC100041057
ENSMUSG00000105263	1.891233216	
ENSMUSG00000105703	1.94042054	
ENSMUSG00000056498	2.013178057	Tmem154
ENSMUSG00000096979	2.563330811	
ENSMUSG00000110386	3.068260462	
ENSMUSG00000107379	4.018599416	
ENSMUSG00000085847	5.160990991	
ENSMUSG00000062611	5.1763928	
ENSMUSG00000058126	5.25145588	
ENSMUSG00000059751	7.594289745	
ENSMUSG00000060795	15.19528034	
ENSMUSG00000052469	15.22625097	Tcp10c
ENSMUSG00000059146	15.48020207	Ntrk3
ENSMUSG00000099913	16.01724418	
ENSMUSG00000034362	16.10432819	Csta1
ENSMUSG00000039814	16.18389028	Xkr5
ENSMUSG00000081078	16.38075379	
ENSMUSG00000073130	16.6843199	Gm1141
ENSMUSG00000061833	16.72269745	
ENSMUSG00000101645	16.99566079	Ctnnd1
ENSMUSG00000091898	17.0054444	Tnnc1
ENSMUSG00000026220	17.00664001	Slc16a14
ENSMUSG00000096718	17.02526929	Zfp781
ENSMUSG00000105820	17.3691121	
ENSMUSG00000092283	17.43077974	
ENSMUSG00000031283	17.501563	Chrdl1
ENSMUSG00000080374	17.74496542	
ENSMUSG00000101397	17.79153262	Mug-ps1
ENSMUSG00000095937	17.89871939	
ENSMUSG00000049555	18.09021599	Tmie
ENSMUSG00000080977	18.09372441	
ENSMUSG00000092029	18.26756917	
ENSMUSG00000096805	18.68315867	
ENSMUSG00000082718	19.59083141	
ENSMUSG00000096712	20.18197979	
ENSMUSG00000047643	20.35459859	
ENSMUSG00000094421	20.36414539	
ENSMUSG00000053830	22.8410883	
ENSMUSG00000104953	23.54925218	
ENSMUSG00000096617	23.93668179	

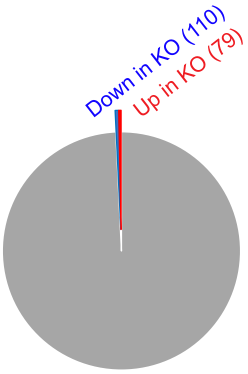

GO Biological process

Genes	cholesterol biosynthetic process	secondary alcohol biosynthetic process	sterol biosynthetic process	cholesterol metabolic process	sterol metabolic process	secondary alcohol metabolic process	alcohol biosynthetic process	steroid biosynthetic process	organic hydroxy compound biosynthetic process	lipid biosynthetic process	steroid metabolic process	farnesyl diphosphate metabolic process	alcohol metabolic process	small molecule biosynthetic process	isoprenoid biosynthetic process	organic hydroxy compound metabolic process
Msmo1	■	■	■	■	■	■	■	■	■	■	■		■	■		■
Cyp51	■	■	■	■	■	■	■	■	■	■	■		■	■		■
Fdft1	■	■	■	■	■	■	■	■	■	■	■	■	■	■	■	■
Hsd17b7	■	■	■	■	■	■	■	■	■	■	■		■	■		■
Sc5d	■	■	■	■	■	■	■	■	■	■	■		■	■		■
Idi1	■	■	■	■	■	■	■	■	■	■	■		■	■	■	■
Hmgcs1	■	■	■	■	■	■	■	■	■	■	■	■	■	■	■	■
Fdps	■	■	■	■	■	■	■	■	■	■	■	■	■	■	■	■
Abcg1	■	■	■	■	■	■	■	■	■	■	■		■	■		■
Pltp							■		■					■		■
Apoc2										■				■		■
Lpl										■				■		■
Insig	■	■	■	■	■	■		■	■	■	■			■		■
Cd244							■		■				■	■		
Trib3										■				■		
Sgms1										■						
Pik3cb										■						
Nanp														■		
Pdgfb										■						

Supplementary Figure 3. Differentially expressed genes in CD1d-KO vs. WT pMacs. Left table show genes ID and symbols as well as the fold change for differentially expressed genes between WT and KO pMacs. A fold change cut-off of 1.5 and adjusted p-value cut off of 0.01 were applied. Top right, show numbers of significantly up- or down-regulated genes. Right table shows GO terms and input genes for significantly changed pathways.

Supplementary Figure 4

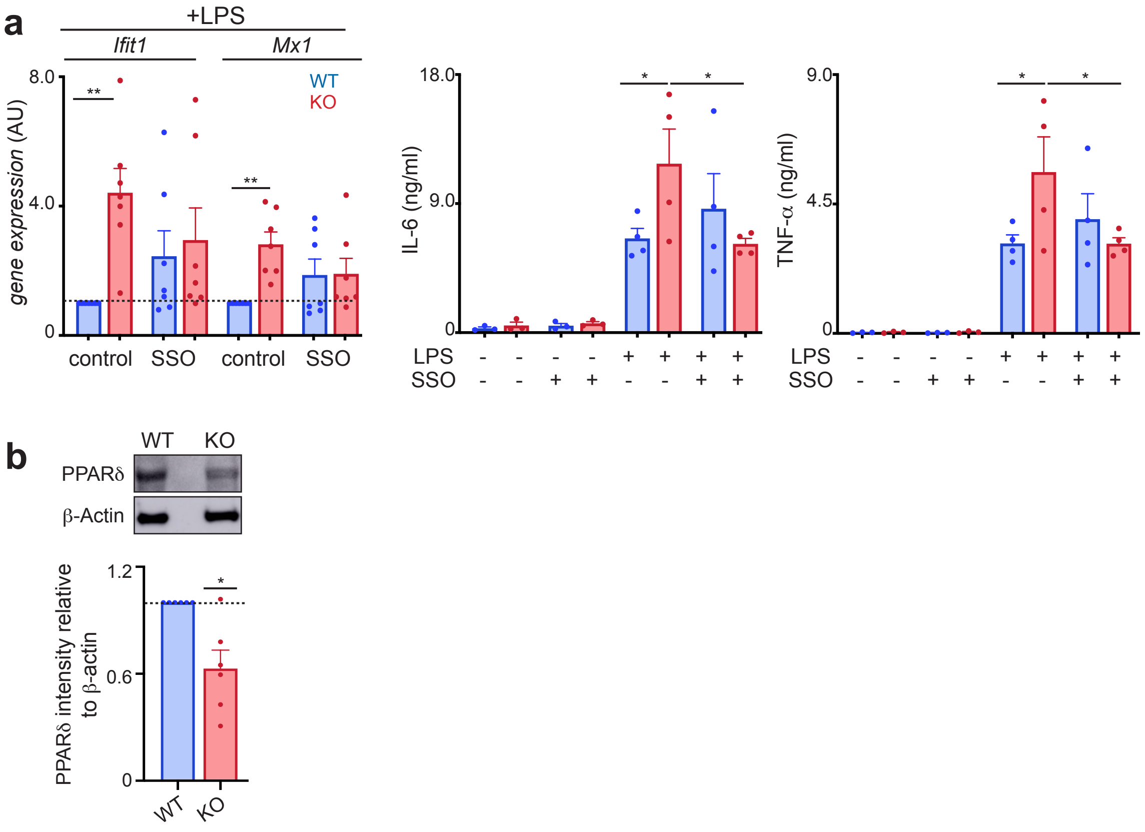

Supplementary Figure 4. CD1d links metabolism and immunity in macrophages. **(a)** BMDCs were cultured in the presence (or absence, control) of SSO overnight prior to stimulation with LPS. Expression of the depicted genes (left, n=7) or cytokine secretion (right, n=3-4) are shown. Bars represent mean +/- SEM; *p<0.05; **p<0.01 2-way ANOVA. **(b)** Western-blots showing total PPARδ and β-actin in WT and CD1d-KO BMDCs. Levels of PPARδ were quantified and related to β-actin (n=6). Bars represent mean +/- SEM; *p<0.05 one-sample t-test. Values for n represent biologically independent samples (as shown by the number of data points in each graph). n= cells isolated from individual mice. Source data are provided as a Source Data file

Supplementary Figure 5

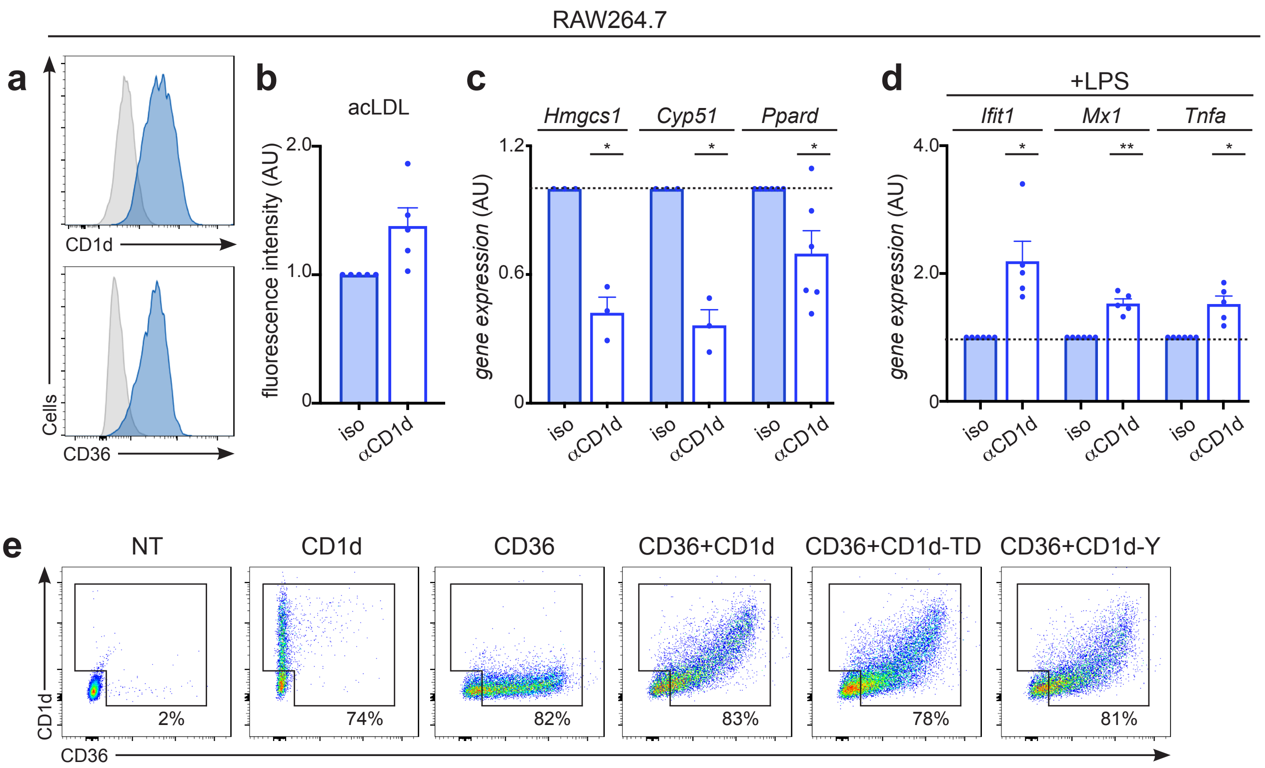

Supplementary Figure 5. CD1d regulates CD36-dependent lipid uptake. (a-d) RAW264.7 cells were treated with αCD1d antibody or isotype control (iso) as indicated. **(a)** Flow cytometry panel showing expression of CD1d and CD36; **(b)** Uptake of DiI-acLDL (n=5); **(c)** Relative expression of the depicted genes (n=3-6); **(d)** Expression of the depicted genes after LPS stimulation (n=5). Bars represent mean +/- SEM; *p<0.05, **p<0.01 one-sample t-test. **(e)** HEK293T cells were transiently transfected with the indicated constructs to express CD1d, mutant CD1d (CD1d-TD or CD1d-Y) and/or CD36. Surface expression of CD1d and CD36 was measured by flow cytometry. Values for n represent biologically independent samples (as shown by the number of data points in each graph). Source data are provided as a Source Data file

Supplementary Figure 6

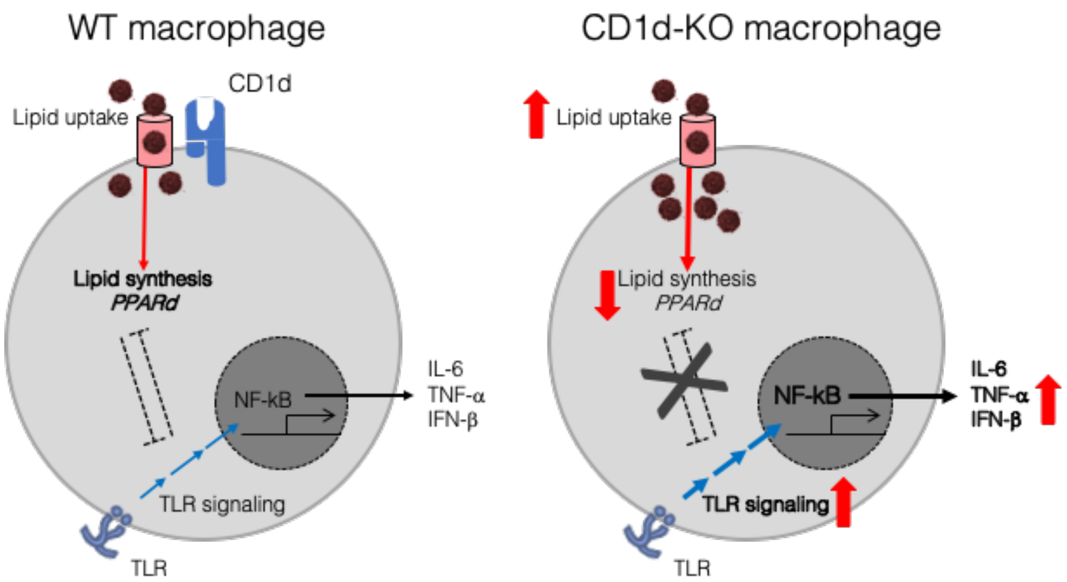

Supplementary Figure 6. Schematic model of the CD1d-dependent regulation of metabolism and immunity in macrophages. CD1d-deficient macrophages display an increased lipid uptake and transcriptional downregulation of metabolic enzymes involved in lipid synthesis pathways as well as the transcription factor PPARδ. These metabolic alterations underpin increased responses of CD1d-KO cells to TLR stimulation, resulting in increased TLR signalling and cytokine production. Red arrows represent pathways up- or down-regulated in CD1d-KO cells vs. WT controls.

Supplementary Table 1. Primers used for qPCR

Target	Forward Sequence	Reverse Sequence
Cd36	ATGGGCTGTGATCGGAACTG	TTTGCCACGTCATCTGGGTTT
Cyp51a1	CTGCCCGCTGGAGCGAAAAG	CACAGGTGTTGTCAGCCGACC
Gapdh	AACGACCCCTTCATTGAC	TCCACGACATACTCAGCA
Hmgcs1	GGAAGCCTTTGGGGACGTTA	ACACTCCAACCCTCTTCCCT
Ifit1	CTGAGATGTCACTTCACATGGAA	GTGCATCCCCAATGGGTTCT
Il6	CAGAGGATACCACTCCCAACA	TCCAGTTTGGTAGCATCCATC
Lpl	GGGAGTTTGGCTCCAGAGTTT	TGTGTCTTCAGGGGTCCTTAG
Msra	TGGAGGAGAGAATCGAAAGCA	CTGGACTGACGAAATCAAGGAA
Mx1	GACCATAGGGGTCTTGACCAA	AGACTTGCTCTTTCTGAAAAGCC
Nrlh3	CTGATTCTGCAACGGAGTTGT	GACGAAGCTCTGTCGGCTC
Nrlh2	CCACCATCGAGATCATGTTG	TCTCGTGGTTGTAGCGTCTGG
Plin1	GGGACCTGTGAGTGCTTCC	GTATTGAAGAGCCGGGATCTTTT
Pltp	CGCAAAGGGCCACTTTTACTA	GCCCCCATCATATAAGAACCAG
Ppara	ACTCCACCTGCAGAGCAACCA	TAFATCTCCTGCAGTAGCGGG
Ppard	CCCTGGCAAAGCATTTGTAT	AATCCTTGGCCCTCTGAGAT
Pparg	TTGAGCCCAAGTTCGAGTTTGCTG	ATTCTAGAGCCCGCAGAATGGTGT
Srebf1	ACAGTGACTTCCCTGGCCTAT	GCATGGACGGGTACATCTTCAA
Srebf2	GCAGCAACGGGACCATTCT	CCCCATGACTAAGTCCTTCAACT
Tlr4	TGGCTGGTTTACACATCCATCGGT	TGGCACCATTGAAGCTGAGGTCTA
