# Supplementary material for: CD1d-dependent rewiring of lipid metabolism in macrophages regulates innate immune responses
Source: Nat Commun. 2022 Nov 7;13:6723. doi: 10.1038/s41467-022-34532-x (PMC9640663; doi:10.1038/s41467-022-34532-x)
Supplement: Supplementary file 3 — Reporting Summary [file 41467_2022_34532_MOESM3_ESM.pdf]

nature portfolio

Corresponding author(s): Patricia Barral

Last updated by author(s): 2022/10/27

Reporting Summary

Nature Portfolio wishes to improve the reproducibility of the work that we publish. This form provides structure for consistency and transparency in reporting. For further information on Nature Portfolio policies, see our Editorial Policies and the Editorial Policy Checklist.

Statistics

For all statistical analyses, confirm that the following items are present in the figure legend, table legend, main text, or Methods section.

n/a	Confirmed	
☐	☒	The exact sample size (*n*) for each experimental group/condition, given as a discrete number and unit of measurement
☐	☒	A statement on whether measurements were taken from distinct samples or whether the same sample was measured repeatedly
☐	☒	The statistical test(s) used AND whether they are one- or two-sided<br>*Only common tests should be described solely by name; describe more complex techniques in the Methods section.*
☒	☐	A description of all covariates tested
☐	☒	A description of any assumptions or corrections, such as tests of normality and adjustment for multiple comparisons
☐	☒	A full description of the statistical parameters including central tendency (e.g. means) or other basic estimates (e.g. regression coefficient) AND variation (e.g. standard deviation) or associated estimates of uncertainty (e.g. confidence intervals)
☐	☒	For null hypothesis testing, the test statistic (e.g. *F*, *t*, *r*) with confidence intervals, effect sizes, degrees of freedom and *P* value noted<br>*Give P values as exact values whenever suitable.*
☒	☐	For Bayesian analysis, information on the choice of priors and Markov chain Monte Carlo settings
☒	☐	For hierarchical and complex designs, identification of the appropriate level for tests and full reporting of outcomes
☐	☒	Estimates of effect sizes (e.g. Cohen's *d*, Pearson's *r*), indicating how they were calculated

Our web collection on statistics for biologists contains articles on many of the points above.

Software and code

Policy information about availability of computer code

Data collection	Flow cytometry: FACSDiva<br>Western-blot: Membranes were imaged in an Amersham Imager 600<br>Real-time PCR system: ABI7900HT (Applied Biosystems)<br>LC-MS/MS for free cholesterol and cholesterol esters was performed on a Nexera liquid chromatography system (Shimadzu) coupled to an API 4000 qTrap mass spectrometer (Sciex).<br>Fluorescent lipid uptake was measured in an EnSight Mulitmode Microplate Reader (PerkinElmer)<br>Confocal microscopy: ZeissUprightLSM710<br>RNAsequencing: Illumina HiSeq 2500
Data analysis	General analyses: FlowJo (BD, version 10.3) to analyze FACS data (https://www.flowjo.com/solutions/flowjo/downloads); Statistical analyses were performed using Prism Graphpad (version 9.1.0) (https://www.graphpad.com/scientific-software/prism/); Fiji/ImageJ (version:2.1.0/153c; https://imagej.net/software/fiji/) was used for image analyses.<br>RNAseq analyses: Fastq files were trimmed using Cutadapt (version 1.9.1) before being aligned to and quantified against ensemble GRCm38 release 86 of the mouse genome (http://www.ensembl.org/Mus_musculus/Info/Index) with RSEM (version 1.3.0)/STAR(version 2.5.2a).<br>The raw counts were then imported into R/Bioconductor (version 3.6.0). DESeq2 (version 1.24.0) was used to estimate different size factors between samples. PANTHER (http://www.pantherdb.org) was used for pathway analyses

For manuscripts utilizing custom algorithms or software that are central to the research but not yet described in published literature, software must be made available to editors and reviewers. We strongly encourage code deposition in a community repository (e.g. GitHub). See the Nature Portfolio guidelines for submitting code & software for further information.

Data

Policy information about availability of data

All manuscripts must include a data availability statement. This statement should provide the following information, where applicable:

- Accession codes, unique identifiers, or web links for publicly available datasets
- A description of any restrictions on data availability
- For clinical datasets or third party data, please ensure that the statement adheres to our policy

Sequencing data associated with this paper has been submitted to NCBI's GEO repository under the accession number GSE215837. Source data are provided with this paper. Mouse genome GRCm38: http://www.ensembl.org/Mus_musculus/Info/Index

Human research participants

Policy information about studies involving human research participants and Sex and Gender in Research.

Reporting on sex and gender	N/A
Population characteristics	N/A
Recruitment	N/A
Ethics oversight	N/A

Note that full information on the approval of the study protocol must also be provided in the manuscript.

Field-specific reporting

Please select the one below that is the best fit for your research. If you are not sure, read the appropriate sections before making your selection.

☒ Life sciences ☐ Behavioural & social sciences ☐ Ecological, evolutionary & environmental sciences

For a reference copy of the document with all sections, see nature.com/documents/nr-reporting-summary-flat.pdf

Life sciences study design

All studies must disclose on these points even when the disclosure is negative.

Sample size	No sample-size calculation was performed for in vivo or in vitro experiments. Sample size was determined on the basis of prior knowledge of variability of similar experiments in our laboratory as well as previous studies: Blanc et al, PLOSBiol 2011; York et al, Cell 2015; Yue et al, PNAS 2005; Saez de Guinoa et al, EMBO reports 2017; Cui et al, Sci Adv 2020; Li et al, Cell Research 2019; Kozicky & Sly, Methods Mol Biol 2019.
Data exclusions	No data were excluded
Replication	All experiments were repeated at least twice yielding similar results.
Randomization	Mice chosen for in vivo analyses were not random and experimental groups were matched for age and gender. Experimental samples of different experimental groups/conditions were processed and analyzed side by side with their respective control samples.<br>For some studies (e.g., ELISA, Western blots, stimulations), conditions/treatments were sometimes performed in different well placements
Blinding	Investigators were not blinded during experiments because no subjective process is included in all the analyses of the experimental data in this study

Reporting for specific materials, systems and methods

We require information from authors about some types of materials, experimental systems and methods used in many studies. Here, indicate whether each material, system or method listed is relevant to your study. If you are not sure if a list item applies to your research, read the appropriate section before selecting a response.

Materials & experimental systems

n/a	Involved in the study
	☒ Antibodies
	☒ Eukaryotic cell lines
☒	☐ Palaeontology and archaeology
	☒ Animals and other organisms
☒	☐ Clinical data
☒	☐ Dual use research of concern

Methods

n/a	Involved in the study
☒	☐ ChIP-seq
	☒ Flow cytometry
☒	☐ MRI-based neuroimaging

Antibodies

Antibodies used	All antibodies, clones, providers and dilutions are included in the methods section. Catalogue number for specific antibodies are labeled with #.<br>Flow cytometry: the following anti-mouse antibodies were used (all from Biolegend unless specified otherwise, all used at 1:200 dilution): CD1d (1B1, #123510), CD11b (M1/70, #101226), CD11c (N418, #B202912), CCR7 (4B12, #120105), CD45.1 (A20, #110732), CD45.2 (104, #109806), CD24 (M1/69, #101824), CD40 (3/23, #124621), CD117 (2B8, #105811), CD80 (16-10A1, #104734), CD86 (GL-1, #105027), TLR4 (SA15-21, #145403), MHC-I (AF6-88.5, BD Biosciences, # 15807288), MHC-II (M5/114.15.2, #107605), CD69 (H1.2F, #104505), CD36 (HM36, #102612), MSR1 (1F8C33, #154710).<br>Western-blot: The following antibodies were used for western blot (all from Cell Signalling unless specified otherwise): b-actin (8H10D10, 1:3000, #3700S), ERK (L34F12, 1:1000, #4696S), p-ERK (9101, 1:1000, #9101S), p38 (D13E1, 1:1000, #8690S), p-p38 (28B10, 1:1000, #9216S), p65 (D14E12, 1:1000, #8242S), p-p65 (93H1, 1:1000, #3033S), CD36 (PA5-33291, ThermoFisher, 1:1000, #PA5-33291), anti-mouse IgG (Poly4053, Biolegend, 1:3000, #405301), anti-rabbit IgG (sc-2955, Santa Cruz Biotechnology, 1:3000, #sc-2357), PPARd (ab23673, Abcam, 1:500, #ab23673).<br>Blocking: CD1d (19G11, InVivoMab, #BE0000, 2ug/ml), CD36 (JC63.1, Abcam, #ab23680, 5ug/ml)<br>Microscopy: CD1d (1B1, Biolegend, #123521, 1:100), CD36 (EPR6573, Abcam, #ab133625, 1:100)<br>PLA: CD1d (K253, Biolegend, #140805, 1:100), CD36 (EPR6573, Abcam, #ab133625, 1:100)
Validation	All antibodies are commercially available and have been validated by the manufacturer and have been widely cited. Validation data / citations can be found on the manufacture website by searching the antibody catalog number provided above

Eukaryotic cell lines

Policy information about cell lines and Sex and Gender in Research

Cell line source(s)	RAW264.7 and HEK293T cells were obtained from the Francis Crick Institute Cell Services Facility and were originally purchased from the ATCC: HEK293T – ATCC catalogue number CRL-3216; Raw 264.7 – ATCC catalogue number TIB-71
Authentication	No authentication was performed
Mycoplasma contamination	Cell lines tested negative for mycoplasma
Commonly misidentified lines (See ICLAC register)	No misidentified cell lines were used in this study

Animals and other research organisms

Policy information about studies involving animals; ARRIVE guidelines recommended for reporting animal research, and Sex and Gender in Research

Laboratory animals	CD1d-KO (B6.129S6-Del(3Cd1d2-Cd1d1)1Sbp/J and CD1dflox x PGKCre), WT C57BL/6, congenic CD45.1 or CD45.1/CD45.2 WT C57BL/6 mice were bred and maintained in individually ventilated cages under specific pathogen-free conditions at the Francis Crick Institute or King's College London. All mice were housed under a 12-hour light/12-hour dark cycle with ad libitum access to food and water, at a temperature of 19-21°C and humidity of 45-65%. Age- and sex-matched mice between 8 and 16 weeks of age were used in the experiments.
Wild animals	The study did not involve wild animals.
Reporting on sex	Male and female mice were used
Field-collected samples	No field collected samples were used in the study
Ethics oversight	All animal experiments were approved by the Francis Crick Institute and the King's College London's Animal Welfare and Ethical Review Body and the United Kingdom Home Office.

Note that full information on the approval of the study protocol must also be provided in the manuscript.

Flow Cytometry

Plots

Confirm that:

☒ The axis labels state the marker and fluorochrome used (e.g. CD4-FITC).

☒ The axis scales are clearly visible. Include numbers along axes only for bottom left plot of group (a 'group' is an analysis of identical markers).

☒ All plots are contour plots with outliers or pseudocolor plots.

☒ A numerical value for number of cells or percentage (with statistics) is provided.

Methodology

Sample preparation	Sample preparation is described in the methods.
Instrument	LSR-II or LSRFortessa for data acquisition or FACSAria II for sorting
Software	Flowjo
Cell population abundance	Peritoneal macrophages were sorted from chimeric mice. Purity after sorting >95%. Example data is included in Sup Fig 1
Gating strategy	FMO (fluorescence minus one) and unstained controls are included for each experiment. Examples of gating strategies are included in Sup Fig 1a and 1d

☒ Tick this box to confirm that a figure exemplifying the gating strategy is provided in the Supplementary Information.
